# The role of cognitive rehabilitation in people with type 2 diabetes: A study protocol for a randomized controlled trial

**DOI:** 10.1371/journal.pone.0285553

**Published:** 2023-05-15

**Authors:** Heather Cuevas, Alexa K. Stuifbergen, Robin C. Hilsabeck, Adam Sales, Shenell Wood, Jeeyeon Kim

**Affiliations:** 1 School of Nursing, The University of Texas at Austin, Austin, Texas, United States of America; 2 Department of Neurology, Dell Medical School, Austin, Texas, United States of America; 3 Mathematical Sciences, Worcester Polytechnic Institute, Worcester, Massachusetts, United States of America; PLOS: Public Library of Science, UNITED KINGDOM

## Abstract

Today, the prevalence of cognitive dysfunction and the prevalence of diabetes are increasing. Research shows that diabetes increases cognitive impairment risk, and cognitive impairment makes diabetes self-management more challenging. Diabetes self-management, essential to good glycemic control, requires patients to assimilate knowledge about their complex disease and to engage in activities such as glucose self-monitoring and the management of their medications. To test a comprehensive cognitive rehabilitation intervention—the Memory, Attention, and Problem-Solving Skills for Persons with Diabetes (MAPSS-DM) program. Our central hypothesis is that participants who take part in the MAPSS-DM intervention will have improved memory and executive function, increased use of compensatory cognitive skills, and improved self-management. We will also explore the role of glucose variability in those changes. This is a randomized controlled trial. Sixty-six participants with cognitive concerns and type 2 diabetes will be assigned to either the full MAPSS-DM intervention or an active control. Participants will use continuous glucose monitoring pre- and post-intervention to identify changes in glycemic variability. All participants will also be evaluated systematically via questionnaires and neuropsychological tests at three timepoints: baseline, immediately post-intervention, and 3 months post-intervention. This study will fill an important gap by addressing cognitive function in the management of diabetes. Diabetes is related to accelerated cognitive aging, cognitive deficits are related to poorer self-management, and improvements in cognitive performance as a result of cognitive rehabilitation can translate into improved performance in everyday life and, potentially, diabetes self-management. The results of the proposed study will therefore potentially inform strategies to support cognitive function and diabetes self-management, as well as offer new mechanistic insights into cognitive function through the use of continuous glucose monitoring.

**Trial registration**: This study has been registered at ClinicalTrials.gov (NCT04831775).

## Introduction

In 2021, it was estimated that 537 million adults were living with diabetes (DM) worldwide, and among those who have DM by age 45 years, DM is correlated with increased odds of accelerated cognitive decline [1, 2]. Furthermore, DM is a significant risk factor for dementia and Alzheimer’s Disease, with cognitive changes beginning early in DM’s development [3, 4]. In obesity and insulin resistance, before development of overt type 2 DM (T2DM), changes in brain activation patterns as well as brain structure have been seen [4–6]. This indicates that in a number of people who are diagnosed with T2DM, negative shifts in cognitive function may have already begun. Nevertheless, although pathologic brain changes related to T2DM have been described [7, 8], much of this information is of limited use to persons actually living with T2DM who need effective strategies to maximize their cognitive functioning in diabetes self-management (DM-SM); indeed research explicating how DM-SM is affected by cognitive function is limited. In other chronic illnesses, interventions to alter lifestyle factors through comprehensive cognitive rehabilitation have been more effective at delaying cognitive decline than have pharmaceuticals, and we expect that the same will be the case for DM [9–12].

In individuals with chronic illnesses, cognitive rehabilitation training can improve efficacy for the use of new cognitive skills, resulting in improved neuropsychological functioning. In the Advanced Cognitive Training for Independent and Vital Elderly study (N = 2,802), such training increased engagement and improved memory in the elderly [13]. Improvement in Memory with Plasticity-Based Adaptive Cognitive Training, one of the more extensive trials examining if a cognitive training intervention could improve cognitive function, showed improved memory, processing speed, and increased self-reported quality of life [14]. However, these studies did not test comprehensive cognitive rehabilitation interventions that combine computer training and practice of compensatory cognitive strategies in T2DM. Some have argued that multimodal cognitive training can produce more robust results than single-domain training [15, 16], and we propose such an intervention here: Memory, Attention, and Problem-Solving Skills for Persons with Diabetes (MAPSS-DM).

The MAPSS-DM is based on the Memory, Attention and Problems Solving Skills for persons with Multiple Sclerosis (MAPSS-MS), which was tested in a fully powered multisite randomized controlled trial (RCT) (N = 183) with a follow-up at 6 months to determine whether improvements on neuropsychological tests were sustained, and whether they affected daily cognitive function [11]. In the MAPSS-MS, the intervention group performed significantly better on selected neuropsychological performance measures than the comparison group and had used compensatory strategies more often immediately after the intervention at 3 months and at 6 months.

To understand the relationship between DM and cognitive function, one must consider glucose variability. Several studies have shown that glucose variability affects cognitive function independently of A1C [17–23]. A recent review of the relationship between glucose variability and cognition found that greater glucose variability was negatively associated with cognitive function in persons with DM [19]; A1C accounted for less than 10% of the change in cognition. Continuous glucose monitoring (CGM) systems enable the identification of fluctuations that A1C does not detect. CGM has been used to evaluate the effects of behavioral interventions on glucose control in T1DM, but to date, no studies have explored the impact of glycemic variability on cognitive function in those with T2DM participating in an intervention to improve cognitive function. Our team is currently conducting a field trial to examine relationships among DM-SM, cognitive function, and the use of CGM. In this project, we will use CGM to explore the potential effect of glucose variability on MAPSS-DM responses.

Given the limited work examining the effects of comprehensive cognitive rehabilitation in T2DM, the MAPSS-DM offers a novel online cognitive rehabilitation intervention. Our overall objective is to help those with T2DM maximize cognitive functioning and develop skills to manage cognitive limitations. This study builds on findings from the PI’s prior work in which participants with T2DM indicated that they experienced difficulties with cognitive functioning, were concerned about those symptoms, and rarely used compensatory cognitive strategies [12, 24].

To our knowledge, no comprehensive, nonpharmacologic interventions address cognitive function within T2DM or DM-SM. Comprehensive interventions are needed to teach effective strategies to manage both cognitive problems to support DM-SM and functioning in everyday life. This study will be the first RCT in the development of a program of research to provide an effective, feasible intervention to address the serious problem of cognitive dysfunction that impacts DM-SM. In determining the effects of the MAPSS-DM on biological, cognitive, and DM-SM outcomes, we expect that those participating in the intervention will show enhanced memory and executive function, increased application of compensatory cognitive strategies, and better DM-SM.

## Materials and methods

### Study design and participants

**Fig 1:**
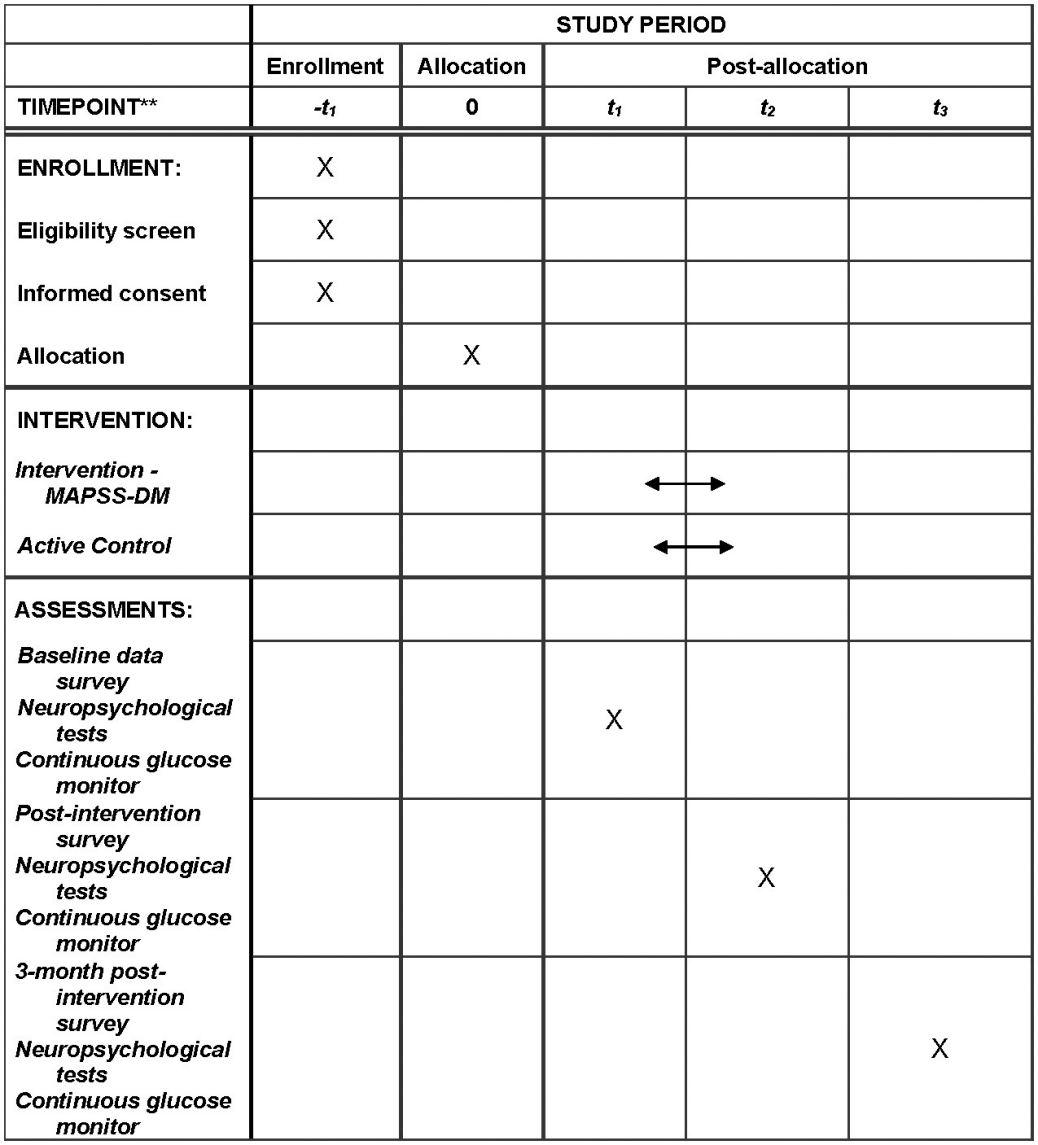
Example template of recommended content for the schedule of enrollment, intervention, and assessments. t_1_: Week 0; t_2_: Week 10; t_3_: Week 22.

A pilot RCT will be conducted with 66 adults with T2DM to evaluate the efficacy of the MAPSS-DM intervention to improve the primary outcome of overall cognitive function (e.g., verbal memory performance, use of cognitive strategies) and DM-SM. We have completed a feasibility study of the intervention using a one-group pretest/posttest design [12, 24], and in the pilot RCT, we will use a two-group design with a longer follow-up (Fig 1). To reduce bias, participants will be randomized 1:1 to the treatment or control group; the data collector will be blinded to group assignment and trained to follow standardized protocols in collecting data; and data collection will be randomly monitored to maintain reliability. Inclusion and exclusion criteria have been designed to control for confounding variables. To ensure treatment fidelity, interventionists will be trained with an intervention manual and will complete checklists after conducting each intervention class. Participants’ receipt of treatment (e.g., time spent online, weekly goals) will also be monitored.

Participation is voluntary and a copy of the consent form will be given to potential participants (paper copy or via email) when they contact study staff regarding participation. The consent form outlines benefits and risks involved in participation, study requirements as well as inclusion/exclusion criteria. Formal consent will be given when the participant digitally signs the consent form on the REDCap survey used for data collection. Participants will have the option to withdraw at any time or to question any part of the procedures. Prior to the start of any study activities, the Institutional Review Board of The University of Texas at Austin approved the project (IRB ID: STUDY00000464).

People with T2DM will be recruited from a multisite endocrinology clinic that oversees the health care of over 2,500 patients with T2DM per year. We have worked with this clinic in prior studies. Information about the study will be provided to patients by the clinic’s health care team, and notices about the study will be posted in the clinic. Recruitment materials will instruct interested potential participants to call or email the PI for information.

Inclusion criteria are as follows: age 45–70 years, having T2DM for at least 2 years, access the Internet via phone or home computer, a score of ≥10 on the Perceived Deficits Questionnaire (PDQ) [25], and an A1C of >7%. Exclusion criteria comprise the following: diagnosis of dementia and/or head injury, significant psychiatric disease, score of >3 on the Mini-Cog [26], inability to speak English. Those who indicate interest in participating in the study will be screened either in person or by phone, using a script with questions to verify inclusion/exclusion. Participants will be asked the PDQ items that assess self-perceived cognitive difficulties, which include questions such as “How often do you lose your train of thought when speaking?” answered on a 5-point scale from 1 = *never* to 5 = *almost always* [26]. To participate, individuals must score at least 10 on the PDQ, demonstrating problems in at least 5 areas. The Mini-Cog will screen potential participants for dementia; a score of ≤3indicates lower likelihood of dementia. For those who meet the inclusion criteria and agree to participate, a meeting will be scheduled at a time and private location convenient to the participant. These eligible individuals will meet with a graduate research assistant or the PI, who will review the study’s purpose and procedures, answer participants’ questions, obtain signed consent forms, and collect baseline data.

**Fig 2:**
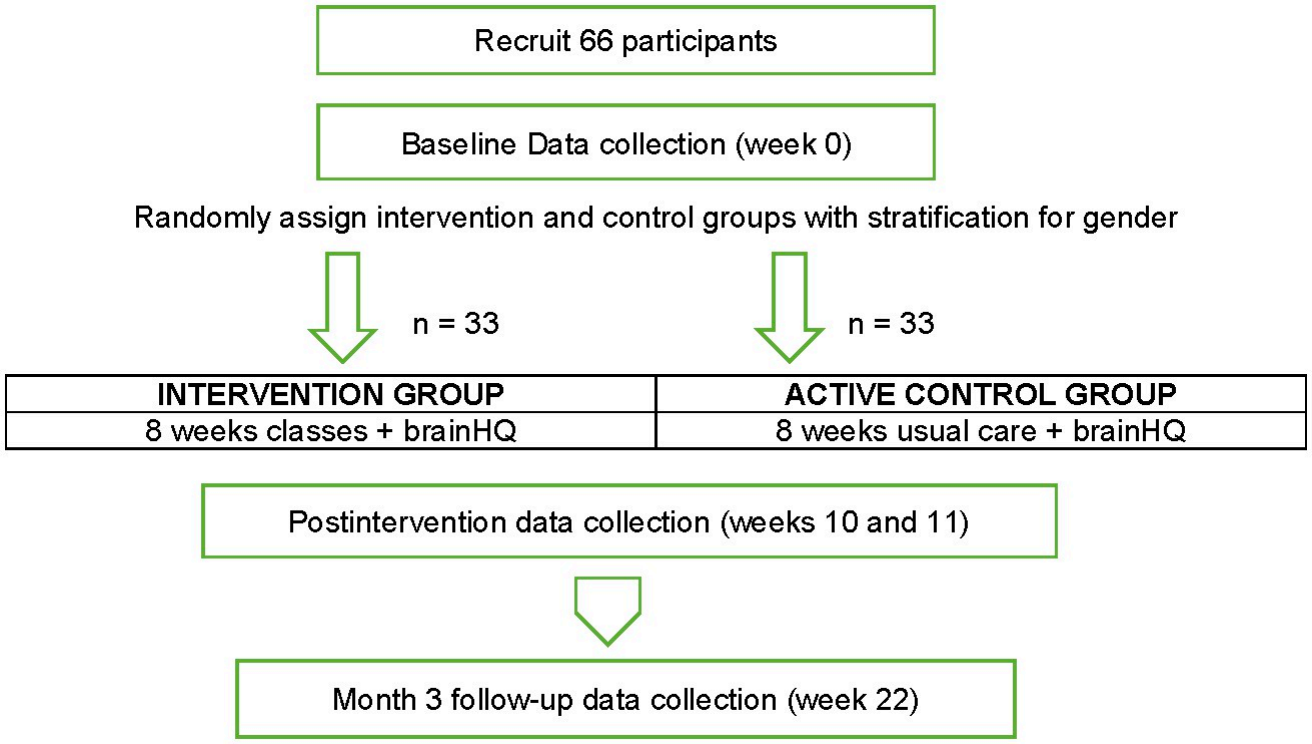
Study diagram.

Three cohorts of 24–26 participants will be recruited during the study, and participants in each cohort will be randomly assigned 1:1 to the intervention or control group using a computer-generated list of random numbers (following baseline testing). The intervention group will be further divided into class sizes of 10–15 participants for instruction (Fig 2). In prior cognitive rehabilitation studies, small intervention classes (n = 10–12) have enhanced self-efficacy through vicarious experience and modeling. In diabetes education, online groups of 10–15 participants have been found effective.

### Sample size calculation

Our power analysis is based on the results of the RCT to evaluate the MAPSS-MS [11], in which the total California Verbal Learning Test was administered at baseline, immediately postintervention, and at 3 and 6 months later. In that study, the within-subjects correlation across timepoints was approximately 0.8, and the effect size (Cohen’s d) was approximately 0.4. For the present project, we used PowerUp! [27] software to estimate the sample size to detect a similar effect at a 0.05 significance level with 80% power. With an intraclass correlation coefficient of 0.8, and assuming that baseline scores explain 64% of individual-level variation, the minimum required sample size is 60. There was no attrition in the MAPSS-MS study; however, we will recruit a larger sample to allow for 5%–10% attrition. With testing on multiple outcomes separately and adjustment for multiplicity using the Benjamini-Hochberg procedure [28], the power to detect effects for at least one outcome is higher than the power for an individual test. Therefore, we consider this power analysis to be conservative.

### Intervention description

The intervention consists of four webinar classes lasting 1 to 1.5 hours, as well as online individual cognitive skills practice done from home. The first two classes will focus on cognitive problems in T2DM and adoption or improvement of cognitive strategies. The third and fourth classes will center on lifestyle modifications to sustain cognitive functioning as well as DM-SM. The format for all four classes will be: (1) introduction (class 1) and briefly reviewing the prior classes’ content; (2) discussion and review of the computer training; (3) discussion and practice of new cognitive strategies; and (4) an additional weekly topic.

#### Online cognitive skills component

Online cognitive skills practice will take place with brainHQ [29]. brainHQ is an interactive and adaptive program accessed via standard web browsers. Study staff will register participants with an anonymous ID number and participants will be able to securely log onto the brainHQ website with their computer, smart phone, or tablet. The website saves each completed training; participants start subsequent trainings wherever they last logged off the site. brainHQ meets the National Academies of Science, Engineering, and Medicine’s prerequisites for these types of programs: (1) transferability to different tasks that measure the same cognitive construct; (2) transferability to real-world tasks; (3) comparison with an active control group; (4) preservation of learned skills; and (5) replication of findings [30]. The intervention group will be asked to practice for 20 minutes, 7 days a week.

#### Active control group

An active control group will be used as recommended by the National Academies [30]. Those randomized to the control group will not attend the classes but will simply receive an email link to the brainHQ site without recommendations for frequency or duration of practice. However, each participant’s practice time will be stored in brainHQ and obtained for analysis. To help with retention, participants will be called or texted weekly. Data will be collected at the same intervals as the intervention group.

### Data collection

#### Survey data

Questionnaire data will be collected from all participants on an iPad and entered into REDCap at baseline (week 0), immediately post-intervention (week 10), and at 3 months post-intervention (week 22). Clinical variables will be gathered by medical record review: current medications, past A1C levels, and comorbidities. Total data collection time will be ~60 minutes. Participants will receive an incentive ($25) for completing the first two data collections and ($50) for the final assessment ($100 total). Survey data will be stored on REDCap and then downloaded into IBM SPSS Statistics version 23 for analysis [31]. Once downloaded all data will be kept securely in UT Box, the University provided cloud storage. UT Box has been approved by the Information Security Office for use with Confidential (formerly known as Category I) university data, including HIPAA data. All intervention participants will be invited to join a focus group after the final data collection to further evaluate the MAPSS-DM intervention.

### Primary outcome measures

#### Assessment of cognitive function

Cognitive function will be assessed using BrainCheck [32], an online platform with established neuropsychological tests that can be administered remotely. Study participants will be sent secure links to the assessment and can complete the tests at their convenience or at the CGM initiation visit to ensure completion. The following tests will be included: (1) Trails A & B; (2) Stroop Color and Word test; (3) Digit-Symbol Substitution; and (4) Immediate and Delayed Recognition tests. A composite score will be calculated to assess global cognitive function (Table 1). Cognitive function data will be stored securely on the BrainCheck server and then downloaded into SPSS for analysis.

**Table 1:** Primary outcome measures.

**Attention**	Trails A & B	Trails A: Participants are asked to select, in order, 25 random numbered circles as fast as possible. Trails B: This requires participants to select alternating numbers and letters, e.g. A, 1, B, 2, etc. The average duration of each trial is measured.
**Executive Function**	Stroop Color and Word test	Measures reaction time required to overcome cognitive interference. The name of a color is displayed in an incongruent color and time taken to name the color of the word is measured. The median duration of incongruent trials is measured.
**Processing Speed**	Digit-Symbol Substitution	Participants match an arbitrary correspondence of symbols to digits. The number of trials correctly completed in 1 minute is assessed.
**Memory**	Immediate Recognition	Ten words are presented, one at a time. Another 20 words are presented, including the 10 previously shown. Participants are asked to identify whether the word appeared previously as quickly as possible.
	Delayed Recognition	At the completion of the other BrainCheck tests, participants must identify whether the word appeared previously or not. Participants will not be able to review the list of words.
**Global Cognitive Function**	Composite Score	A scaled standard score compares the composite score of the above tests to the scores for the same age range. The scaled standard score ranges from 0 to 200, with the average score corresponding to the 50^th^ percentile receiving a score of 100. One standard deviation is equal to 15 points, so, for example, a score of 85 is 1 standard deviation below the mean.
**DM-SM**	Summary of Diabetes Self-Care Activities	18 items: Participants answer “how many days in the last week…” they performed DM-SM (e.g. diet and physical activity). Inter-item correlations range from r = 0.20 to 0.76 for four SDCA subscales; 4-month test–retest reliability ranges from r = -0.05 to 0.78.

#### Assessment of diabetes self-management

The Summary of Diabetes Self-Care Activities [33] will be used to measure diabetes self-management. This is an 18-item survey in which participants answer “How many days in the last week…” with respect to performed DM-SM tasks such as diet management and physical activity. Inter-item correlations range from r = 0.20 to 0.76 for four SDCA subscales; 4-month test–retest reliability ranges from r = -0.05 to 0.78.

#### Assessment of glucose variability

Participants will wear CGM devices for 2 weeks at specific intervals (week 0, week 11, and week 22). Abbott’s FreeStyle Libre Pro CGM system [34] includes a sensor about the size of two stacked U.S. quarters which is worn on the back of the upper arm. The sensor measures participants’ interstitial glucose every minute through a small filament inserted just under the skin. CGM data will be downloaded at each data collection visit. A reader is scanned over the sensor to get a glucose result in about 1 second. The stored data from the monitor will be converted to glucose concentrations using an infrared link to a personal computer and analyzed using CGM software maintained by the research staff. The following glucose composites will be calculated: (1) the overall mean, (2) the proportion of readings indicating hypoglycemia (% <70mg/dL), (3) the proportion of readings indicating hyperglycemia (% >160mg/dL), (4) the proportion of out-of-range readings (% either <70mg/dL or >160mg/dL), (5) the coefficient of variation, (6) the time in range, and (6) the standard deviation of CGM readings [35, 36]. Compared with self-monitoring of blood glucose, 85.5% of FreeStyle Libre Pro CGM readings have been found to be clinically accurate and acceptable [34]. CGM data will be stored in LibreView, a secure web-based data management system and then downloaded into SPSS.

### Secondary outcome measures

#### Assessment of depression

To measure depression, we will use the Center for Epidemiological Studies Depression (CES-D) tool [37]. This is a 20-item survey with a 4-point item response scale from *rarely*/*none of the time* to *most*/*all of the time* on 8 health dimensions: role limitations due to physical problems, social functioning, physical functioning, bodily pain, general mental health, role limitations due to emotional problems, vitality, and general health perceptions. Internal consistency has ranged from 0.85 to 0.91 and test–retest reliability from 0.45 to 0.70.

#### Assessment of self-efficacy

The Diabetes Empowerment Scale–Short Form will be used to measure self-efficacy [38]. This is a brief eight-item tool with responses on a 5-point scale from 1 = *strongly disagree* to 4 = *strongly agree*. Items include statements such as “I believe that I am able to turn my diabetes goals into a workable plan,” and “I know positive ways I cope with diabetes related stress.” Cronbach’s alphas range from -0.81 to 0.96.

#### Assessment of perceived cognitive function

The PROMIS v2.0–Cognitive function scale will be used to assess perceived cognitive function [39]. This 32-item scale measures perceived cognitive deficits in areas including mental acuity, concentration, verbal and nonverbal memory, and verbal fluency. Reliability has been measured at 0.94 and test–retest correlation at 0.83.

#### Demographics

Participants’ characteristics will include age, gender/sex, years with diabetes, ethnicity/race, socioeconomic status, history of diabetes education, and years of education.

### Statistical analysis

We will use multilevel longitudinal models to estimate MAPSS-DM treatment effects on measurements of cognitive function, A1C, and DM-SM over time. These models, generalizations of repeated-measures ANOVA, include every participant in the analysis regardless of missing data and account for the clustering of measurements within participants. We will regress each outcome variable on random intercepts for each participant and effects of the intervention (MAPSS-DM vs. control), interacted with measurement occasion (week 0, 11, or 22), cohort, and baseline covariates; specifically, we will stratify baseline measures of the dependent variables (cognitive function, A1C, and DM-SM), demographics, and other baseline measures of anticipated prognostic value. We will use the models to test for overall treatment effects (p < 0.05), effects at each post-baseline timepoint, and within-subjects effects. Effect sizes will be expressed as Cohen’s d.

CGM data will be downloaded at each data collection visit, and glycemic variability summary statistics for each participant will be calculated at weeks 0, 11, and 22. We will use regression models analogous to those for MAPSS-DM treatment effects on cognitive function, A1C, and DM-SM to estimate the MAPSS-DM’s effect on glucose variability at post-baseline timepoints. We will use causal mediation methods and exploit the longitudinal structure of the data collection to estimate the extent to which glycemic variability mediates the effect of MAPSS-DM on cognitive function. To do so, we will fit two sets of regression models: the first to estimate the effects of MAPSS-DM on each measure of glycemic variability at week 11, and the second to estimate the effects of MAPSS-DM and week 11 glycemic variability on each measure of cognitive function at week 22.

All regression models will be stratified on appropriate baseline measures and covariates. The direct effects of MAPSS-DM on week 22 cognitive function measures—estimated by the coefficients for the MAPSS-DM dummy variables in the second set of regressions—is the extent to which MAPSS-DM would have affected cognitive function, had week 11 glycemic variability been held at the control level for each participant. The average mediated effect of the intervention on cognitive function will be estimated by the sum of the coefficients on the MAPSS-DM dummy variable from the first set of regressions multiplied by the coefficients on the corresponding CGM statistics in the second regression, with Monte-Carlo confidence intervals and statistical tests. This will estimate the average effect on week 22 cognitive function of the changes in week 11 glycemic variability induced by MAPSS-DM—the extent to which MAPSS-DM affects cognitive function by affecting glycemic variability. To address potential differences in cognitive function, A1C, glucose variability, and DM-SM between men and women, we will refit appropriate regression models from the analyses and include interaction terms between intervention group and sex.

### Data and safety monitoring plan

Prior to the start of study activities, approval will be obtained by University’s Institutional Review Board. A Safety Monitoring Committee (SMC) will be the monitoring entity. The SMC will consist of members not involved in the project. The PI, co-Is and consultants will contribute to the study oversight. The SMC will consist of three members, including an MD, with experience in conducting clinical trials for chronic diseases including diabetes and cognitive dysfunction, expertise in biostatistics, and a thorough knowledge of clinical trial ethics and subject protection issues. The SMC responsibilities are to: (1) review the research protocol, informed consent documents and plans for data safety and monitoring; (2) evaluate the progress of the trial, including periodic assessments of data quality and timeliness, recruitment, accrual and retention, participant risk versus benefit, performance of the trial sites, and other factors that can affect study outcome; (3) consider factors external to the study when relevant information becomes available, such as scientific or therapeutic developments that may have an impact on the safety of the participants or the ethics of the trial; (4) review study performance, make recommendations and assist in the resolution of problems reported by the PI; (5) report to NINR on the safety and progress of the trial; and (6) if appropriate, review interim analyses in accordance with stopping rules, which are clearly defined in advance of data analysis and have the approval of the SMC.

Review of participant accrual, adherence to inclusion/exclusion criteria will occur monthly during each recruitment phase to assure that participants meet eligibility criteria and ethnic diversity goals outlined in the grant proposal. Data on adherence to the treatment protocol will be collected monthly by research staff and reviewed by the PI. Participant adherence will be evaluated for (1) group class attendance and the (2) individual practice on the home computer games. If the PI or SMC has concerns about whether compliance has reached a level that might inhibit the ability of the study to test its primary hypotheses, they will suggest a meeting for the study investigators to discuss methods for improving compliance.

Adverse events will be categorized according to the likelihood that they are related to the study intervention. Specifically, they will be labeled either definitely, probably, possibly or unrelated to the study intervention. Adverse events that are unanticipated and/or possibly related to the study intervention will be reported by the PI to the SMC, IRB and NIH within 24 hours of the PI learning of the event. Anticipated adverse events or those unrelated to the study intervention will also be reported to the same individuals/entities. If the PI or other members of the research team become aware of injury or other adverse event, participants will be referred to their physician for follow-up and will be advised not to engage in intervention-related activities until we receive approval from their physician. We will be persistent with following-up with the patient and physician. A standard IRB Adverse Event Report Form will be completed within 15 days of the event. This report will include a full description of the event, including the relationship of the adverse event as not related, possibly related, or definitely not related to the test procedure.

Code numbers will be assigned to each participant. The SPSS file will contain participants’ numerical code and their responses to the survey. The file will be saved onto a password-protected computer owned by the PI. Aggregated, de-identified data will be made available to other researchers upon request to the PI.

### Dissemination

All the requirements will be adhered to regarding registering and reporting our study to ClinicalTrials.gov and the NIH Public Access Policy to submit all accepted peer-reviewed publications that result from this study to the National Library of Medicine’s PubMed Central. Since the proposed project is a randomized controlled trial of a behavioral intervention, the PI is the responsible party who will carry out the tasks and meet the deadlines as defined in the NIH Policy on Dissemination of NIH-funded Clinical Trial Information.

## Discussion

The prevalence of cognitive dysfunction in chronic diseases such as diabetes continues to increase worldwide but tested therapeutic strategies to improve symptoms of cognitive dysfunction in this population are lacking. This adds a burden for patients and families who are already dealing with self-management of a chronic illness, such that the need to develop therapeutic strategies is urgent. In the forthcoming project, we will test a comprehensive cognitive training intervention to determine whether this intervention has the potential to improve both objective and subjective cognitive function and explore possible mechanisms of action such as glucose variability.

In our previous study, postintervention scores improved in all areas of DM-SM and cognitive domains and were statistically significant for diet adherence, t(18) = -2.41, p < 0.05; memory ability, t(18) = 5.54, p < 0.01; and executive function, t(18) = 3.11, p < 0.01. Effect sizes (Cohen’s d) ranged from 0.56 to 0.75. Many participants (58%) stated that the intervention helped their DM-SM, and 75% expressed the desire to keep using cognitive strategies that they had learned [12]. However, participants recommended reducing daily practice time to 20 minutes, 7 days per week. This should help improve recruitment and retention. Although the prior study relied on self-report measures of cognition, this next project will use objective neurocognitive tests. We are including A1C but adding CGM as a measure of glucose variability. An integrative review has found that cognitive dysfunction in those with T2DM can negatively impact DM-SM [40]. In clinical practice, however, cognitive health is often not part of the discussion of diabetes complications [41]. This demonstrates a need for routine screening for cognitive problems and conversations about improving or maintaining cognitive health.

A number of studies have outlined modifiable risk factors to decrease the incidence of cognitive dysfunction [42–44]. Cognitive training offers another type of intervention, and it is important to create shared insights about not only the benefits and drawbacks of cognitive training, but also underlying mechanisms. A systematic review of the effects of RCT behavioral interventions (including 26 cognitive training studies) has found that cognitive training consistently improved verbal immediate recall, cognitive speed, and verbal and visual working memory [45]. Additionally, the cognitive training component of the MAPSS-DM intervention meets the National Academies of Science, Engineering, and Medicine’s requirements for a cognitive training program [30]. The protocol for our intervention is modeled after programs that meet these criteria. The assessment and delivery of the intervention will be completed by specifically trained, experienced registered nurses.

In summary, the objective of this project is to determine whether a comprehensive cognitive rehabilitation intervention will be effective in improving DM-SM, cognitive function, and glucose control. Meanwhile, it will also help us identify how glucose variability is (or is not) a mechanism that influences cognitive function.

## Supporting information

S1 Checklist SPIRIT 2013 checklist: Recommended items to address in a clinical trial protocol and related documents. (DOC)

S1 File Study protocol. (DOCX)
