# Supplementary material for: The role of cognitive rehabilitation in people with type 2 diabetes: A study protocol for a randomized controlled trial
Source: PLoS One. 2023 May 15;18(5):e0285553. doi: 10.1371/journal.pone.0285553 (PMC10184896; doi:10.1371/journal.pone.0285553)
Supplement: S1 File — (DOCX) [file pone.0285553.s002.docx]

Cognitive Training for Diabetes Self-Management

## Sponsor:

University of Texas at Austin

## Information provided by (Responsible Party):

University of Texas at Austin

| **Tracking Information** |
| --- |
| **First Submitted Date** |
|  |

| April 2, 2021 |
| --- |
| **First Posted Date** |
| April 5, 2021 |
| **Last Update Posted Date** |
| May 17, 2022 |
| **Actual Study Start Date ^I^** |
| November 1, 2021 |
| **Estimated Primary Completion Date** |
| March 28, 2023 (Final data collection date for primary outcome measure) |
| **Current Primary Outcome Measures**  **(submitted: April 2, 2021)** |
| Change in A1C at week 22 [ Time Frame: Baseline, and week 22 ] Measure of average glucose over 3 months  Change in diabetes self-management adherence at week 22 [ Time Frame: Baseline, week 8, and week 22 ]  Summary of Diabetes Self-Care Activities: 8 items; Brief assessment of diabetes related psychosocial self-eﬃcacy. Responses are made on a 5-point scale (1 = strongly disagree to 4 = strongly agree) to items such as "I believe that I am able to turn my diabetes goals into a workable plan." Cronbach's alphas range from 0.81 to 0.96. Higher scores indicate higher levels of self-management adherence.  Change in glucose variability at week 22 [ Time Frame: Baseline, week 8, and week 22 ]  Continuous Glucose Monitor (CGM): To be worn for 2 weeks at baseline and week 22; The following glucose composites will be calculated: (1) the overall mean, (2) the proportion of readings indicating hypoglycemia (%<70mg/dL), (3) the proportion of readings indicating hyperglycemia (% >160mg/dL),  (4) the proportion of out of range readings (% either <70mg/dL or >160mg/dL) and (5) the SD of CGM glucose readings.  Change in attention, visual scanning and motor speed at week 22 [ Time Frame: Baseline, week 8, and week 22 ]  Symbol Digit Modalities Test: Participants are given a series of symbols and digits and instructed to verbalize the digit associated with each symbol. The number of correct responses in 90 sec constitutes the score, and higher scores reﬂect better cognitive function. |
| **Original Primary Outcome Measures** |
|  |

| Change in perceived cognitive function at week 22 [ Time Frame: Baseline, week 8, and week 22 ]  PROMIS v2.0 - Cognitive Function: 32 items; assess patient-perceived cognitive deﬁcits including the areas of mental acuity, concentration, verbal and nonverbal memory, and verbal ﬂuency. Reliability has been measured at 0.94 and test-retest correlation at 0.83. Higher scores indicate more perceived diﬃculty with cognitive function. |
| --- |
| **Original Secondary Outcome Measures** |
| *Same as current* |
|  |
|  |
| **Descriptive Information** |
| **Brief Title** |
| Cognitive Training for Diabetes Self-Management |
| **Oﬃcial Title** |
| Cognitive Training for Diabetes Self-Management |
| **Brief Summary** |
| The overall objective of this study is to determine the eﬀects of a comprehensive cognitive rehabilitation intervention on biological, cognitive, and diabetes self-management outcomes. |
| **Detailed Description** |
| Aim 1: Test the eﬃcacy of the MAPSS-DM intervention for improving cognitive function, A1C, and DM-SM. Based on preliminary data, the working hypothesis is that compared with the control group, persons who receive the intervention will have improved memory, executive function, and perceived cognitive function, |

| greater use of cognitive strategies, and improved DM-SM immediately post-intervention and at three and six-months post-intervention.  Aim 2: To explore changes in glycemic variability and their association with changes in cognitive function. The working hypothesis here is that MAPSS-DM participants will exhibit less glycemic variability post- intervention as compared with baseline and glycemic variability will mediate improvements in cognitive test performance. |
| --- |
| **Study Type** |
| Interventional |
| **Study Phase** |
| Not Applicable |
| **Study Design** |
| Allocation: Randomized  Intervention Model: Parallel Assignment  Masking: Double (Investigator, Outcomes Assessor) Masking Description:  The intervention will be delivered by a research assistant and outcome data collection will be done by two other research assistants  Primary Purpose: Treatment |
| **Condition** |
| Diabetes Mellitus, Type 2 |
| **Intervention** |
| Behavioral: Memory, Attention, and Problem Solving Skills for Diabetes  The intervention is composed of 4 small-group webinar classes and home-based individual online cognitive skills practice and will be held over 8 weeks. The classes will be taught by a GRA. Classes 1 & 2 will focus on common cognitive problems in T2DM and strategies to improve cognitive skills. Classes 3 & 4 focus on lifestyle changes to support cognitive functioning and DM-SM skills. Each online class will follow the same format: (1) introduction/revisiting content from the previous class; (2) review of progress on computer exercises; (3) practicing cognitive strategies in class; and (4) a weekly topic. The GRA will also prescribe exercises for the following weeks.  Behavioral: Brain Games  The computer-training component uses a model for cognitive training that adapts to the user through an integrated hierarchical structure. The BrainHQ website houses the interactive program that runs on standard web browsers. Participants will only need a computer, smart phone, or tablet with Internet access to securely log onto the website. Each participant will be registered by the project staﬀ using |

| anonymous ID numbers that will allow unlimited access during the study. The website stores each session completed, and participants can start subsequent sessions wherever they stopped the last time logged on.  Other Name: BrainHQ |
| --- |
| **Study Arms** |
| Experimental: Memory, Attention, and Problem Solving Skills for Diabetes  The intervention is composed of 4 small-group webinar classes and home-based individual online cognitive skills practice over 8 weeks. Classes 1 & 2 will focus on common cognitive problems in T2DM and strategies to improve cognitive skills. Classes 3 & 4 focus on lifestyle changes to support cognitive functioning and DM-SM skills. The computer-training component uses a model for cognitive training that adapts to the user through an integrated hierarchical structure. The BrainHQ website houses the interactive program that runs on standard web browsers. Each participant will be registered by the project staﬀ using anonymous ID numbers that will allow unlimited access during the study. The website stores each session completed, and participants can start subsequent sessions wherever they stopped the last time logged on. The intervention group will be asked to practice 20 minutes, 7 days a week.  Intervention: Behavioral: Memory, Attention, and Problem Solving Skills for Diabetes  Active Comparator: Brain Games Only  An active control group will be used. The diﬀering variable between the two groups is the class sessions. Those randomized to the control group will only receive a link to the BrainHQ games site. A speciﬁc amount of practice will not be prescribed, but the frequency and duration of participant's practice will be obtained from BrainHQ. Participants will receive a weekly phone call to maintain connection to the study. Data collection will be on the same schedule as the intervention group.  Intervention: Behavioral: Brain Games |
| **Publications *** |
| *Not Provided* |
|  |
| *** Includes publications given by the data provider as well as publications identiﬁed by ClinicalTrials.gov Identiﬁer (NCT Number) in Medline.** |
|  |
|  |
| **Recruitment Information** |
| **Recruitment Status** |
| Recruiting |

| **Estimated Enrollment (submitted: April 2, 2021)** |
| --- |
| 70 |
| **Original Estimated Enrollment** |
| *Same as current* |
| **Estimated Study Completion Date** |
| March 28, 2023 |
| **Estimated Primary Completion Date** |
| March 28, 2023 (Final data collection date for primary outcome measure) |
| **Eligibility Criteria** |
| Inclusion Criteria:  age 50 years old or greater T2DM diagnosis for 2 years access to phone and Internet  Score of ≥10 on the Perceived Deﬁcits Questionnaire (PDQ) A1C of >7%.  Exclusion Criteria:  a diagnosis of dementia/head injury score of >3 on the Mini-Cog  inability to speak English, and T1DM diagnosis |
| **Sex/Gender** |
| **Sexes Eligible for Study:**  All |
| **Ages** |
| 50 Years and older (Adult, Older Adult) |
| **Accepts Healthy Volunteers** |
| Yes |
| **Contacts** |
| **Contact: Heather E Cuevas, PhD** |

| [hcuevas@mail.nur.utexas.edu](mailto:hcuevas@mail.nur.utexas.edu) |
| --- |
| **Listed Location Countries** |
| United States |
| **Removed Location Countries** |
|  |
|  |
|  |
| **Administrative Information** |
| **NCT Number** |
| NCT04831775 |
| **Other Study ID Numbers** |
| 00000464 |
| **Has Data Monitoring Committee** |
| Yes |
| **U.S. FDA-regulated Product** |
| **Studies a U.S. FDA-regulated Drug Product:**  No  **Studies a U.S. FDA-regulated Device Product:**  No |
| **IPD Sharing Statement** |
| **Plan to Share IPD:**  No  **Plan Description:**  Only de-identiﬁed quantitative and qualitative data will be available to other qualiﬁed researchers upon request from the PI two years after completion of the study. After review of the purpose of the data request to make sure it is consistent with the original project goals and veriﬁcation that the request meets IRB approval, de-identiﬁed data may be shared with researchers two years after completion of the study. It is recognized that the government reserves the right to identify repositories for submission of data for archive as stated in the grant instructions. |
| **Current Responsible Party** |
|  |

| University of Texas at Austin |
| --- |
| **Original Responsible Party** |
| [*Same as current*](https://clinicaltrials.gov/ct2/history/NCT04831775?V_1=View&StudyPageTop) |
| **Current Study Sponsor** |
| University of Texas at Austin |
| **Original Study Sponsor** |
| [*Same as current*](https://clinicaltrials.gov/ct2/history/NCT04831775?V_1=View&StudyPageTop) |
| **Collaborators** |
| *Not Provided* |
| **Investigators** |
| **Principal Investigator:**  Heather E Cuevas, PhD  The University of Texas at Austin |
| **PRS Account** |
| University of Texas at Austin |
| **Veriﬁcation Date** |
| May 2022 |
|  |
|  |
